# Venetoclax in Combination with Decitabine for Relapsed T-Cell Acute Lymphoblastic Leukemia after Allogeneic Hematopoietic Cell Transplant

**DOI:** 10.1155/2018/6092646

**Published:** 2018-08-26

**Authors:** Leena T. Rahmat, Anna Nguyen, Haifaa Abdulhaq, Sonam Prakash, Aaron C. Logan, Gabriel N. Mannis

**Affiliations:** ^1^Department of Hematologic Malignancies and Blood and Marrow Transplant, University of California, San Francisco, CA, USA; ^2^Department of Pathology, University of California, San Francisco, CA, USA; ^3^Department of Hematology and Oncology, University of California, San Francisco, CA, USA

## Abstract

Long-term disease-free survival in adults with T-cell acute lymphoblastic leukemia (T-ALL) remains poor, particularly after relapse, with few available salvage options. Preclinical data suggest that inhibition of the antiapoptotic protein BCL-2 (B-cell lymphoma 2) either alone or in combination with other agents, may be a unique therapeutic approach for the treatment of T-ALL. We present a case of a young male with T-ALL, relapsed after allogeneic hematopoietic stem cell transplant, who achieved a second complete remission following salvage therapy with combined venetoclax and decitabine. Assessment of measurable residual disease by next generation sequencing showed no evidence of residual disease of a sensitivity of 1 × 10^−6^. While the combination of venetoclax and hypomethylating agents has shown promise in the treatment of relapsed/refractory AML, and to our knowledge, this is the first report of this combination demonstrating clinical activity in relapsed/refractory T-ALL.

## 1. Introduction

T-cell acute lymphoblastic leukemia (T-ALL) is an aggressive hematologic malignancy that accounts for approximately 25% of all adult ALL cases [1]. Despite an improved cure rate in the pediatric ALL population, long-term disease-free survival in adults remains poor [2]. Outcomes for patients with relapsed or refractory T-ALL are particularly dismal, with few available therapeutic options. Recent preclinical studies have demonstrated that inhibition of the antiapoptotic protein BCL-2 (B-cell lymphoma 2) may be a novel therapeutic strategy for the treatment of T-ALL, either alone or in combination with other agents [3, 4]. Venetoclax (ABT-199) is a highly specific BCL-2 inhibitor, currently approved for the treatment of chronic lymphocytic leukemia. Genetic studies in T-ALL have also recognized recurrent somatic alterations involved in DNA methylation and posttranslational histone modifications, suggesting a potential rationale for the combination of venetoclax and hypomethylating agents [5].

## 2. Case Presentation

A 20-year-old male with no past medical history presented with acute hypoxic respiratory failure requiring intubation. CT scans revealed a 9.1 × 7.3 cm mediastinal mass encasing the aortic arch with extension into the lower neck resulting in tracheal deviation. Laboratory evaluation demonstrated a white blood cell count of 2.5 × 10^9^/L with 78% blasts on differential, hemoglobin 7.1 g/dL, and platelets 51 × 10^9^/L. Bone marrow evaluation revealed a hypercellular marrow with 98% blasts by morphology. By flow cytometry, blasts expressed CD34, CD117, CD33, CD38, CD56, and CD7 and lacked expression of myeloperoxidase (MPO) and monocytic markers. A subset of blasts expressed low levels of cytoplasmic CD3 although subsequent assessment by immunohistochemistry for CD3 was negative. The blasts were negative for CD2, CD4, CD5, CD8, CD19, and cCD79a. Cytogenetic analysis revealed 10 metaphases with a complex karyotype including rearrangement of chromosome 4, loss of chromosomes 12 and 13, and a rearrangement between chromosome 13 and 1-2 unidentified markers. Molecular testing was positive for a FLT3-ITD mutation. A fine needle aspiration of the mediastinal mass demonstrated acute leukemia with an immunophenotype similar to that of the bone. T-cell gene rearrangement analysis by PCR on the mediastinal biopsy showed no evidence of clonal T-cell gene rearrangement. While it was difficult to assign a definite lineage for this acute leukemia, diagnostic considerations included acute myeloid leukemia (AML), T-ALL, and mixed phenotype acute leukemia T/myeloid (MPAL). To meet criteria for MPAL T/myeloid, blasts must express lineage-defining markers for both T and myeloid lineages [6]. This acute leukemia lacked MPO as well as monocytic markers and therefore did not meet criteria for the myeloid component of MPAL T/myeloid. While flow cytometry demonstrated weak cytoplasmic CD3 on the blasts suggestive of T-lineage differentiation, this could not be confirmed by immunohistochemical stains. Additionally, the blasts lacked expression of CD2, CD4, CD5, CD8, and CD1a. Therefore, a diagnosis of AML, NOS was initially favored.

The patient was induced with daunorubicin in combination with high-dose cytarabine and achieved complete remission (CR). He was consolidated with 2 cycles of high-dose cytarabine and in light of the FLT3-ITD mutation, sorafenib was added. Because his leukemia was considered “poor-risk” given the extramedullary disease at presentation, complex karyotype, and FLT3-ITD mutation, he underwent a 7/10 haploidentical allogeneic hematopoietic cell transplant (alloHCT) from his father in first remission. Conditioning included fludarabine, cyclophosphamide, and low-dose total body irradiation as per the standard Hopkins regimen [7]. Prior to alloHCT, multiparameter flow cytometry from the University of Washington showed no evidence of measurable residual disease (MRD). Posttransplant immunosuppression consisted of cyclophosphamide, tacrolimus, and mycophenolate mofetil. A bone marrow biopsy and restaging PET CT at approximately day +60 confirmed an ongoing CR and 100% donor chimerism in the CD3, CD14/15, and CD19 compartments. For posttransplant maintenance, he received 6 cycles azacitidine (given daily for 5 days at 32 mg/m^2^ in 28-day cycles)—initiated at approximately day +100—followed by maintenance sorafenib 200 mg twice daily. His tacrolimus was discontinued at approximately 6 months posttransplant with no evidence at any point of either acute or chronic graft-versus-host disease (GVHD).

At 13 months posttransplant, he developed progressive neutropenia. Bone marrow evaluation revealed relapsed leukemia with 42% blasts expressing a slightly different immunophenotype than that of his original disease (CD117-negative and CD5-positive). Immunohistochemical stains on the core biopsy demonstrated that blasts were positive for CD34, TdT, CD5, and CD7, with a small subset that was weakly positive for CD3. Cytogenetic studies demonstrated a complex karyotype similar to that of his original leukemia. FLT3-ITD PCR was negative but extended mutational testing—not previously performed—revealed mutations in FBXW7, NOTCH1, and EZH2, all of which are recurrently mutated in T-ALL [8]. Additionally, bone marrow chimerism studies showed for the first time a decline in CD3 donor chimerism from 100% to 91%.

Based on emerging data to support the use of venetoclax in T-ALL, he began salvage therapy with venetoclax (given daily, initially at 800 mg then dose reduced to 400 mg due to the interaction with his azole for fungal prophylaxis) in combination with decitabine (given daily for 5 days at 20 mg/m^2^ in 28-day cycles). After 2 cycles, his peripheral blood counts had normalized, formal CR criteria were met, and a restaging bone marrow evaluation demonstrated no morphologic evidence of residual leukemia. MRD assessment—again performed via multiparameter flow cytometry at the University of Washington—also showed no evidence of persistent disease. CD3 chimerism in the bone marrow was restored to 100% donor.

In light of the lymphoid origin of his relapsed leukemia, next generation sequencing (NGS) was performed (ClonoSEQ, Adaptive Biosciences) on his relapse specimen, identifying a dominant T-cell receptor (TCR) clone comprising 8.249% of total nucleated cells. We retrospectively also assessed his original diagnostic bone marrow specimen and found that the same TCR clone was present in 11% of total nucleated cells. MRD assessment from a bone marrow specimen after 2 cycles of venetoclax and decitabine—also via ClonoSEQ—showed no detectable residual leukemia at a sensitivity of 1 leukemic cell per 10^6^ cells. He received a total of 5 cycles of decitabine and venetoclax, with intermittent dose interruptions of venetoclax due to neutropenia. He remained MRD negative via multiparameter flow cytometry and ClonoSEQ and subsequently underwent a second haploidentical alloHCT with fludarabine, cyclophosphamide, and low-dose TBI conditioning. Decitabine and venetoclax were discontinued shortly prior to the second haploidentical alloHCT. He achieved full donor chimerism at day +18 posttransplant and continues in follow-up.

## 3. Discussion

We present the case of a young male—initially diagnosed with AML, NOS but which, in retrospect, was T-ALL—who achieved a first complete remission following induction chemotherapy and subsequently underwent haploidentical alloHCT. He relapsed 13 months later with disease that was immunophenotypically and genetically consistent with T-ALL. He achieved a second complete remission following 2 cycles of the BCL-2 inhibitor venetoclax in combination with the hypomethylating agent (HMA) decitabine, with no evidence of MRD by either multiparameter flow cytometry or NGS. He remained in remission following completion of 5 cycles of decitabine and venetoclax and subsequently underwent a second haploidentical alloHCT. At the time of this writing, he remains in remission and continues in routine posttransplant follow-up.

While the combination of venetoclax and HMAs have shown significant promise in the treatment of relapsed/refractory AML, to our knowledge, this is the first report of the combination showing clinical activity in relapsed/refractory T-ALL [9]. *In vitro* studies demonstrate that some T-ALL cell lines are highly sensitive to BCL-2 inhibition [4]. One subgroup of T-ALL, early T-cell progenitor (ETP) T-ALL, has historically been characterized by treatment-resistance and high risk for relapse. Preclinical data show that ETP T-ALL is highly BCL-2 dependent and is sensitive to both *in vitro* and *in vivo* treatment with venetoclax [10]. Early T-cell precursors have high expression of BCL-2 but this gradually decreases during normal T-cell differentiation. Variations in venetoclax sensitivity could therefore be mediated in part by varying stages of differentiation arrest between different subtypes of T-ALL [4].

Studies by both Bogenberger et al. [11] and Tsao et al. [12] initially showed that the combination of azacitidine and BCL-2 inhibition synergistically induced apoptosis in AML cells. As monotherapy, HMAs have limited data to support their use in ALL, although several *in vitro* studies suggest that epigenetic regulation may be important in the pathogenesis of ALL [5, 13]. Preclinical studies have shown that promoter regions of several genes associated with biological and prognostic significance in ALL—including the tumor suppressor gene, FHIT (fragile histidine triad), and ERG isoforms—are methylated in ALL and can be demethylated with exposure to hypomethylating agents [14, 15]. The limited clinical data supporting the use of HMAs in ALL include a case report of an adult patient with concurrent MDS and B-ALL who achieved a morphologic remission of both ALL and MDS with azacitidine monotherapy, as well as an older study evaluating the efficacy of single agent azacitidine in a variety of acute leukemias [16]. This study found that only 2 of 23 patients with ALL achieved a CR.

In light of this patient's prior posttransplant exposure to HMAs, it seems unlikely that an HMA alone would have been sufficient to induce an MRD-negative second remission. Although impossible to know if the disease may have responded to venetoclax monotherapy, we hypothesize that—similar to what has been described in the AML setting—venetoclax and HMAs are likely to work synergistically to eradicate relapsed T-ALL [17]. If proven to be effective in larger, prospective studies, the combination of venetoclax and HMAs would be particularly attractive in the posttransplant relapse setting given its relatively nontoxic profile.

## 4. Conclusion

The prognosis of relapsed/refractory T-ALL is poor, with few available therapeutic options. Preclinical studies have shown both that BCL-2 inhibitors may be effective in T-ALL and that epigenetic regulation plays a role in ALL pathogenesis. In addition to the emerging data suggesting mechanistic synergy between BCL-2 inhibition and HMA therapy in AML, this case provides rationale for further study of venetoclax and HMAs in relapsed T-ALL, particularly in the posttransplant setting.
